# Large-scale synthesis and exciton dynamics of monolayer MoS_2_ on differently doped GaN substrates

**DOI:** 10.1515/nanoph-2023-0503

**Published:** 2023-11-22

**Authors:** Pengcheng Jian, Xueqing Cai, Yongming Zhao, Dongyan Li, Zheng Zhang, Weijie Liu, Dan Xu, Wenxi Liang, Xing Zhou, Jiangnan Dai, Feng Wu, Changqing Chen

**Affiliations:** Wuhan National Laboratory for Optoelectronics, Huazhong University of Science and Technology, Wuhan 430074, China; State Key Laboratory of Materials Processing and Die & Mould Technology, School of Materials Science and Engineering, Huazhong University of Science and Technology, Wuhan 430074, China

**Keywords:** MoS_2_/GaN heterostructure, large-scale synthesis, CVD, substrate-induced doping, exciton dynamics

## Abstract

Mixed dimensional van der Waals heterostructure based on layered two-dimensional molybdenum disulfide (MoS_2_) interfaced to gallium nitride (GaN) has attracted tremendous attention due to its unique properties and application in novel electronic, optoelectronic, and quantum devices. However, developing facile synthesis methods and insights into the exciton dynamics for this system still remains a major challenge. Here, a simple and cost-effective method is demonstrated for large-scale synthesis of monolayer MoS_2_ on differently doped GaN substrates. A mixed aqueous solution of Na_2_MoO_4_ and NaOH is spin-coated on GaN and sulfurated in one step by chemical vapor deposition (CVD). High quality monolayer MoS_2_ nanosheets with side length over 400 μm and surface coverage ratio of more than 90 % are achieved on GaN. Furthermore, the PL intensity, excitonic transition ratios and ultrafast exciton dynamics of MoS_2_ are observed to be largely modulated by the doping type of GaN, owing to substrate-induced doping, which is proved by Raman, PL and transient absorption spectroscopy. Notably, p-GaN can attract electrons from monolayer MoS_2_ and weaken its intrinsic n-doping, thereby facilitating higher PL intensity as well as longer exciton lifetime, while n-GaN provides strong n-doping and generates opposite effect. This work hereby presents a pathway for large-scale synthesis of MoS_2_/GaN heterostructures and further understanding of their charge transfer properties and exciton dynamics, which should inspire their applications for optoelectronic devices.

## 1 Introduction

Atomically thin two-dimensional (2D) transition metal dichalcogenides (TMDCs), especially monolayer molybdenum disulfide (MoS_2_), has attracted extensive interest due to its unique electrical, mechanical, optical and chemical properties, which make it promising candidate for various applications in electronics, sensors, energy storage, and optoelectronics [1–6]. Moreover, the absence of dangling bonds in this 2D layered material facilitates its heterogeneous integration with traditional three-dimensional (3D) semiconductors, utilizing the benefits of both 2D layered materials and well established 3D thin film semiconductors [7]. For instance, these mixed dimensional van der Waals heterostructures hold the potential for significantly improved responsivity and active wavelength range, which provides unique opportunities towards highly efficient *p*-*n* junction solar cells, ultrabroadband photodetectors, light-emitting diodes (LEDs), and high power devices [8–11].

As a typical III–V compound, gallium nitride (GaN) is widely known as one of third-generation semiconductors extensively used in electronic and optoelectronic devices, depending on its wide direct bandgap (3.4 eV), high electron mobility as well as excellent chemical and thermal stability [12, 13]. Furthermore, these unique properties and compatibility with mature process semiconductor technology make GaN an ideal 3D material to construct heterostructures with 2D layered materials such as MoS_2_. Notably, both GaN and MoS_2_ belong to the hexagonal crystal system, processing similar lattice constants (3.19 Å for GaN, 3.16 Å for MoS_2_), together with a small discrepancy on the thermal expansion coefficients (3.95 × 10^−6^ K^−1^ for GaN, 4.92 × 10^−6^ K^−1^ for MoS_2_) [14, 15]. The almost perfect lattice matching and small difference in thermal expansion coefficient are of great importance to guarantee high quality heterostructures. These attractive heterostructures meet the demands of new electronic and optoelectronic devices with multifunctionality and provide new platforms for theoretical studies in fundamental physics in recent years [15–18]. However, none of these studies has focused on the varying substrate engineering effects on the doping and exciton dynamics of MoS_2_ due to differently doped GaN, which is crucial to understand the optical properties systematically and improve the device performance in optoelectronic and light-harvesting application.

So far, the fabrication of MoS_2_/GaN heterostructures has largely depended on top-down approaches, by employing mechanical exfoliation or wet chemical transfer [19–21]. However, these methods are evidently tedious and severely limit the size and heterointerface quality. In contrast, the bottom-up growth methods can achieve large-scale and high quality MoS_2_ nanosheets on GaN directly in a simpler way. Among the various growth techniques, chemical vapor deposition (CVD) is a comparatively simple route and most used for the direct synthesis of MoS_2_, but it is still challenging to obtain large-area and uniform monolayer MoS_2_ single-crystal nanosheets on GaN substrates using this method currently. Several strategies have been developed for the direct synthesis of MoS_2_/GaN heterostructures, such as confined-space CVD growth using the molybdenum-oxide-based precursors [22], as well as seed-assisted CVD method with the help of perylene-3,4,9,10-tetracarboxylic acid tetrapotassium salt (PTAS) seeds [23]. However, both of them are in demand of complicated treatment process and obtain MoS_2_ nanosheets with the size below 100 μm only in a small area. Recently, vapor–liquid–solid (VLS) growth has been demonstrated as an attractive approach for high quality uniform MoS_2_ isolated flakes, using non-volatile alkali molybdates instead, which possess extremely low vapor pressure and produce low-melting-temperature compound to facilitate further deposition [24, 25]. Moreover, the alkali molybdates possess the advantage to be prepared in stable aqueous solutions that can either be uniformly deposited or patterned by mold on large wafers via spin-coating [26, 27]. It is therefore highly promising toward the large-scale synthesis of MoS_2_/GaN heterostructures with better controllability, improved yield and reproducibility.

Herein, we report a simple and inexpensive approach for large-scale synthesis of MoS_2_/GaN heterostructures by spinning a mixed solution of Na_2_MoO_4_ and NaOH on GaN substrates followed by CVD sulfurization. In this way, triangle-shaped monolayer MoS_2_ single-crystal nanosheets with coverage of nearly 90 % and a side length of more than 400 μm can be obtained on differently doped GaN substrates. Meanwhile, a series of characterizations were conducted, suggesting that the as-grown MoS_2_ nanosheets on GaN are of high quality and uniformity. Moreover, Raman and photoluminescence (PL) spectroscopy, together with transient absorption (TA) spectroscopy were employed, in order to investigate the influence of the differently doped GaN on the exciton dynamics of MoS_2_. Changes in doping of MoS_2_ were observed, due to the substrate-induced doping effect. Additionally, the PL emission, excitonic transition ratios and ultrafast exciton dynamics of MoS_2_ were also greatly affected by the underlying differently doped GaN. Our results provide insight into the large-scale synthesis, substrate engineering effects on the doping and exciton dynamics of differently doped MoS_2_/GaN heterostructures, which should inspire their application in the field of optoelectronic devices.

## 2 Experimental section

### 2.1 Synthesis of MoS_2_/GaN heterostructures

GaN was grown by metal–organic chemical vapor deposition (MOCVD) on c-plane sapphire substrates, during which the SiH_4_ gas and Cp_2_Mg sources were used for n-type and p-type doping, respectively. The specific epitaxial structures of differently doped GaN are shown in Figure S1, including Si doping concentration around 4 × 10^18^ cm^−3^ for n-GaN, Mg doping concentration around 2 × 10^19^ cm^−3^ for p-GaN and unintentional doping for u-GaN. The as-prepared GaN substrates were ultrasonically cleaned in acetone, absolute ethanol, and deionized water successively for 15 min, then blown dry with a nitrogen gas gun before the growth process of MoS_2_.

**Figure 1:**
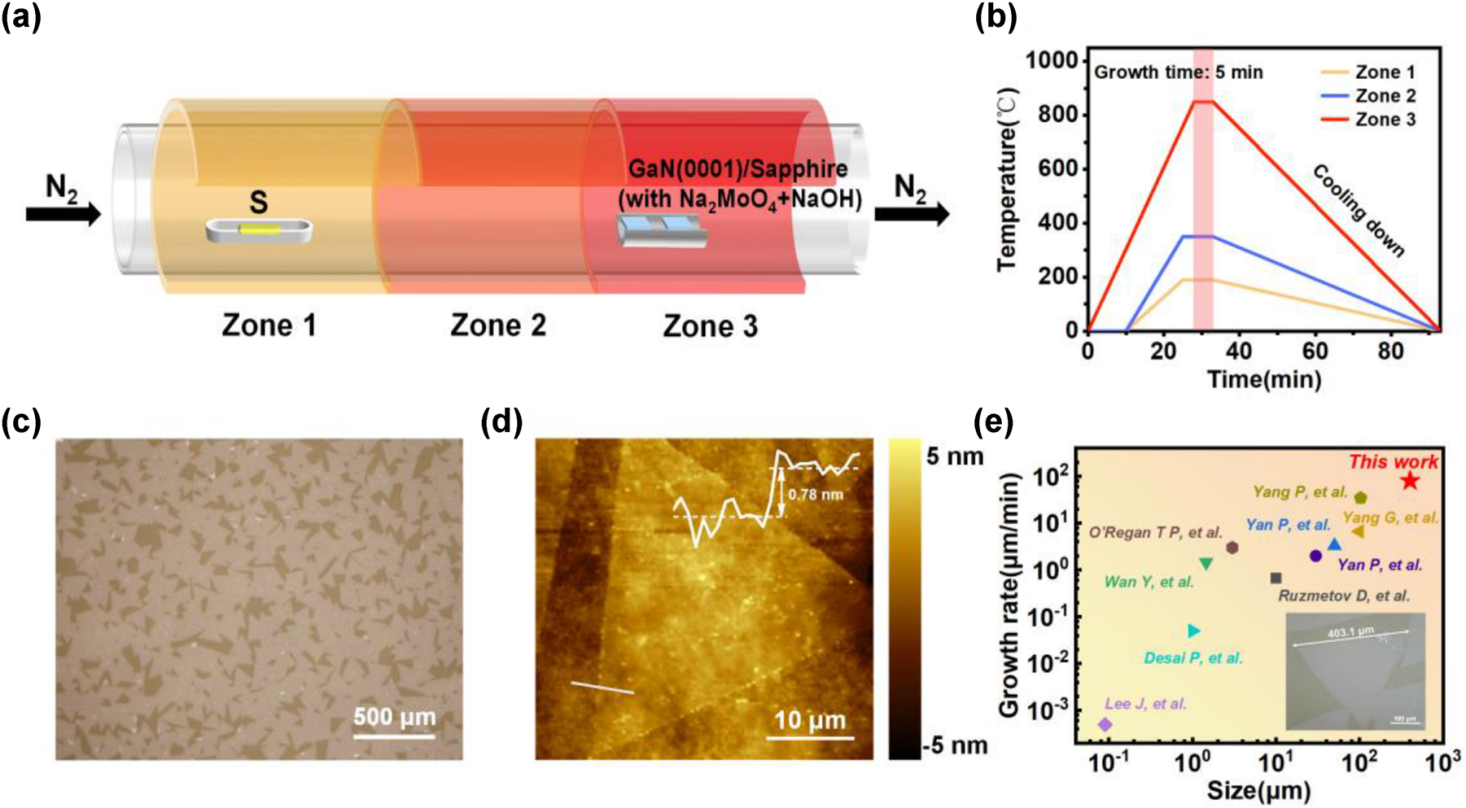
Growth of monolayer MoS_2_ on GaN(0001)/sapphire substrates. (a) Schematic illustration of the CVD setup for the growth of monolayer MoS_2_ on GaN(0001)/sapphire substrates. (b) Temperature programming process of S source and Mo source. (c) Optical microscopy image of the as-grown MoS_2_ nanosheets. (d) AFM image of the MoS_2_ nanosheet. Inset is a height profile along the white line. (e) The maximum size and growth rate of monolayer MoS_2_ on GaN synthesized by direct CVD growth as reported in other articles [15, 23, 31–37]. The magnified optical microscopy image of a large single crystal MoS_2_ nanosheet is presented as the inset (the scale bar is 100 μm).

As for the growth of MoS_2_, sodium molybdate (Na_2_MoO_4_) was employed as a precursor owing to its low melting point, which can easily transform into a molten liquid intermediate state at a relatively lower temperature. Meanwhile, sodium hydroxide (NaOH) was involved as a promoter to further facilitate the mobility and lateral growth of the molten liquid intermediates [28]. 155 mg Na_2_MoO_4_ (99 %, aladdin) and 30 mg NaOH (99 %, aladdin) were firstly dissolved in 100 mL de-ionized water to obtain mixture solutions. These solutions were then spin-coated (1000 rpm for 30s, followed by 2000 rpm for 90 s) on the GaN substrates pretreated by O_2_ plasma, aiming to achieve a homogeneous distribution of precursor, which is important for obtaining large-area and uniform MoS_2_ nanosheets. Notably, the O_2_ plasma treatments were maintained for 300 s at a power of 50 W and an O_2_ gas-flow rate of 30 sccm using a Plasma Cleaner (Tonson Tec, YZD08-10C). After being completely dried, they were put into a 3-inch three-zone furnace, 50 cm downstream from a ceramic boat carrying 400 mg S powder (99.5 %, aladdin), as shown in Figure 1(a). The three-zone furnace temperature was subsequently risen up to 190/350/850 °C as depicted in Figure 1(b) (with Zone 2 acting as moderate temperature zone to avoid the mutual influence of zone 1 and zone 3) and maintained for 5 min with 250 sccm N_2_ flow in an atmospheric pressure for the growth. Meanwhile, the vapor–liquid–solid (VLS) growth occured, during which Na–Mo–O liquid droplets formed and adsorbed sulfur vapor continuously. These droplets diffused laterally on the substrate and precipitated out MoS_2_ when saturated, finally achieved large-size MoS_2_, as depicted in Figure S2.

### 2.2 Material characterization and optical measurement of MoS_2_/GaN heterostructures

The morphology of as-grown MoS_2_ nanosheets on GaN substrates was characterized using an optical microscope (Sunny Optical Technology, RX50M). The atomic force microscope (AFM) images were characterized with the aid of a Bruker Dimension AFM system (Bruker Dimension Edge). The Raman spectra and PL spectra were carried out by a laser confocal micro-Raman spectrometer (Horiba Jobin-Yvon, LabRAM HR800). Raman spectra was recorded with two accumulations with 15 s accumulation time under the excitation of a 532 nm excited line for all samples. PL spectra was collected under the excitation of a 532 nm and a 325 nm excited line with the integration time of 5 s. The Raman and PL mapping were recorded by a confocal microscope spectrometer (Alpha300 Raman, WITec). The XPS spectra was performed by an X-ray photoelectron spectroscopy (Kratos AXIS SUPRA+), in which the binding energies of the photo-emission spectra was calibrated against the C 1s peak of adventitious carbon at 284.8 eV. The ultrafast exciton dynamics characterization was performed on a transient absorption spectrometer (HELIOS, Ultrafast Systems) pumped by a Ti:sapphire regenerative amplifier (Legend Elite, Coherent) operating at 5 kHz with fundamental wavelength of 800 nm and pulse width of ∼40 fs. For measurements of MoS_2_/GaN heterostructures, the probe beam with a wavelength range spanning over 450–750 nm was focused to a spot with a diameter of ∼3 μm, and the pump beam with a wavelength of 400 nm was focused to a spot with a diameter of ∼5 μm.

## 3 Results and discussion

### 3.1 Large-scale synthesis of monolayer MoS_2_ on GaN substrates

The typical procedure for synthesizing monolayer MoS_2_ nanosheets on GaN substrates was conducted by a NaOH assisted vapor-liquid-solid CVD method, with more details about the specific growth parameters and steps described in the Experimental Section. The morphological characteristics of as-grown MoS_2_ nanosheets on u-GaN substrates were analyzed by optical microscopy (OM) and AFM. From its magnified OM image displayed in Figure 1(c), uniform color contrast can be clearly seen, indicating the uniform thickness and in-plane continuity of the MoS_2_ nanosheets. Large triangular MoS_2_ crystal domains can be distinguished with an average edge length over 200 μm, reaching the surface coverage ratio of more than 90 %. Meanwhile, it is found that the orientation of these MoS_2_ domains is randomly distributed, which can be attributed to the large amount of NaOH introduced [28]. The addition of Na element and hydroxide ions has been proven to reduce the nucleation density, inhibit the vertical crystal growth and facilitate the lateral mobility of the Na–Mo–O liquid droplets during VLS growth, leading to monolayer MoS_2_ domains with large size and random arrangements [25, 29, 30]. The OM images of MoS_2_ nanosheets grown on other doped GaN substrates are shown in Figure S3, indicating similar size and morphology. Figure 1(d) shows the AFM topography image of the synthesized MoS_2_ nanosheet. The height profile provided as inset (extracted from the white line in Figure 1(d)) shows a thickness of 0.78 nm, corresponding to a monolayer. It is also worth mentioning that the maximum side length of triangular MoS_2_ domains can reach up to 403 μm, together with rapid growth rate over 80 μm/min, which surpass the values of previously reported MoS_2_ domains grown on GaN substrates, as compared in Figure 1(e) [15, 23, 31–37].

**Figure 2:**
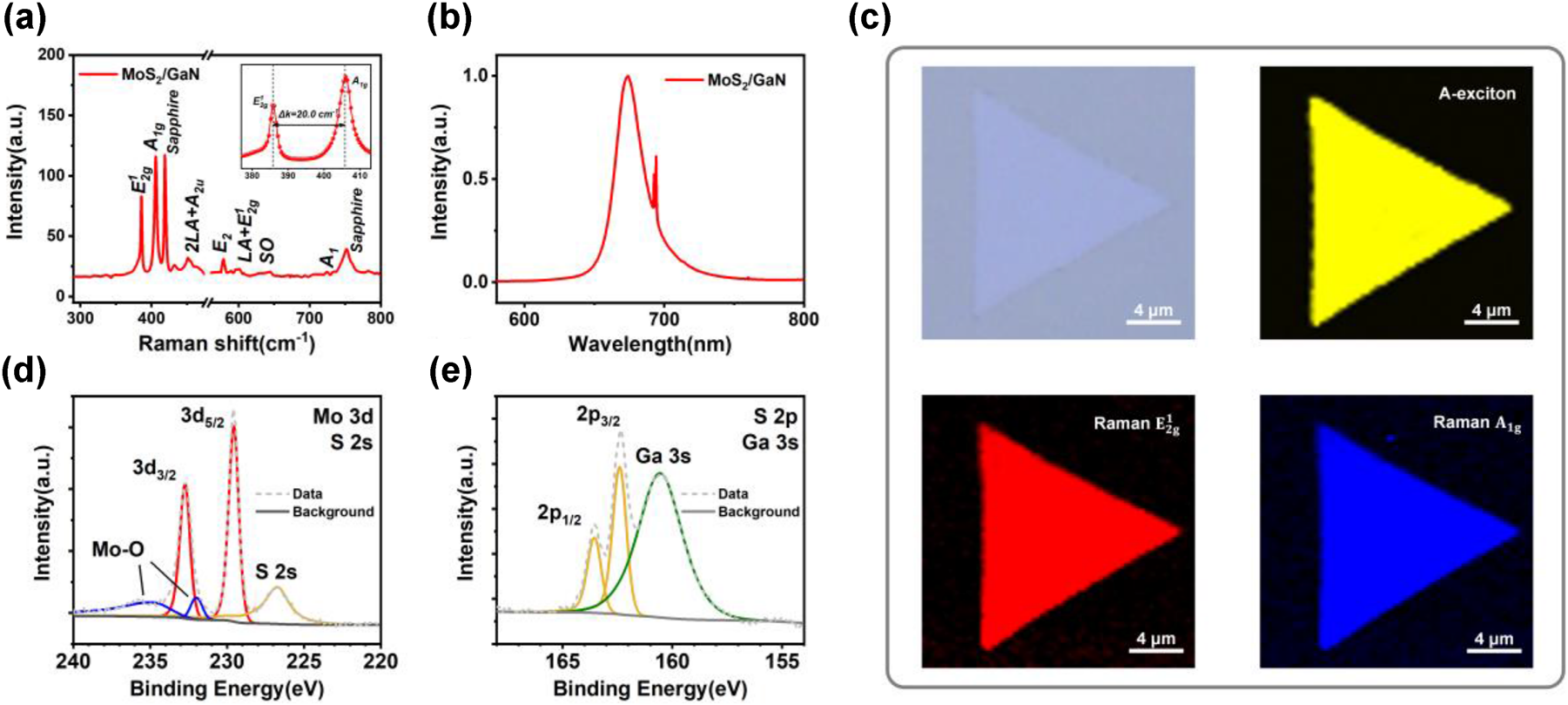
The composition and quality characterizations of the MoS_2_/GaN heterostructures. (a, b) Raman (a) and photoluminescence (b) spectra of monolayer MoS_2_ grown on GaN, respectively. The inset in (a) is a close-up view for the characteristic Raman modes of MoS_2_. (c) Optical microscopy image, PL intensity mapping for A-exciton, and Raman intensity mapping for $E_{2\text{g}}^{1}$ and *A*_1g_ peak of a representative MoS_2_ single crystal. (d, e) Core level XPS spectra and peak fits from (d) Mo *3d* and (e) S *2p* regions for the MoS_2_/GaN heterostructure. Components from S *2s* (MoS_2_) and Mo *3d* (MoS_2_ and MoO_*x*_), together with S *2p* doublet (MoS_2_) and Ga *3s* (GaN) are shown in (d) and (e), respectively.

The composition and crystal quality of the MoS_2_/GaN heterostructures were characterized with Raman, PL, and XPS spectra. Two characteristic Raman modes of MoS_2_ can be observed in the spectra, as shown in Figure 2(a). The *A*_1g_ mode corresponds to the out-of-plane vibration of sulfur atoms and the $E_{2\text{g}}^{1}$ mode associates with the in-plane vibration of Mo and sulfur atoms [38]. For the MoS_2_ nanosheets on u-GaN substrates, the two Raman characteristic peaks are fixed at 385.8 cm^−1^ ( $E_{2\text{g}}^{1}$ ) and 405.8 cm^−1^ (*A*_1g_), with a frequency difference (Δ*k*) ∼20.0 cm^−1^, justifying the monolayer nature of the MoS_2_. Furthermore, the full width at half maximum (FWHM) of the $E_{2\text{g}}^{1}$ peak is 2.3 cm^−1^, which is even better than high-quality MoS_2_ samples reported previously [39]. As for GaN, two typical Raman modes are observed at 578.7 cm^−1^ and 735.6 cm^−1^, representing the *E*_2_ high and *A*_1_ longitudinal optical (LO) phonon modes, respectively. Meanwhile, both the broad Raman mode at 451.0 cm^−1^ and the surface optical (SO) phonon of GaN at 636.6 cm^−1^ can be attributed to the interlayer electron–phonon coupling of the heterostructure [16, 40]. In addition, the PL spectrum in Figure 2(b) demonstrates a sharp peak at 673.0 nm with relatively high intensity and a narrow FWHM (16.8 nm), suggesting the good crystallinity of the monolayer MoS_2_ nanosheets on GaN (the PL spectrum of the substrate is provided in Figure S4 as a comparison). In order to further investigate the uniformity of monolayer MoS_2_ nanosheets on GaN substrates, PL mapping integrated by the intensity of A exciton emission, as well as Raman mappings with the intensity of $E_{2\text{g}}^{1}$ mode and *A*_1g_ mode were conducted, as displayed in Figure 2(c). Clear color contrast across the entire area is manifested, which indicates the uniform thickness and excellent crystallinity of the samples with few structural defects or disorder at the edge.

XPS spectra of the heterostructure are depicted in Figure 2(d), (e) and Figure S5. The Mo^4+^ *3d*_5/2_ and *3d*_3/2_ peaks are located at 229.5 eV and 232.7 eV, while the S^2−^ *2p*_3/2_ and *2p*_1/2_ peaks are located at 162.3 eV and 163.5 eV, respectively, consistent with the corresponding binding energies reported in the literature for MoS_2_ single crystal [41]. Also, the stoichiometric ratio between S and Mo from the intensities of the respective XPS peaks could be calculated as S: Mo = 1.92: 1, further confirming the high quality of MoS_2_ nanosheets with few sulfur vacancies. In addition, the Ga *3s* peak is observed at 160.5 eV (Figure 2(e)), together with Ga *2p*_1/2_ and Ga *2p*_3/2_ peaks at around 1145.4 and 1144.8 eV (Figure S5(a)), which are in line with the XPS spectra of GaN reported previously, further indicating the stability of GaN during the growth of MoS_2_ [42]. Moreover, there is no signature of any chemical state associated with Mo or S in the Ga *3d* core level spectra (Figure S5(b)), suggesting atomically sharp heterointerface, which is clear evidence of a van der waals interaction between MoS_2_ and GaN.

### 3.2 Doping effect and exciton dynamics of monolayer MoS_2_ on differently doped GaN

**Figure 3:**
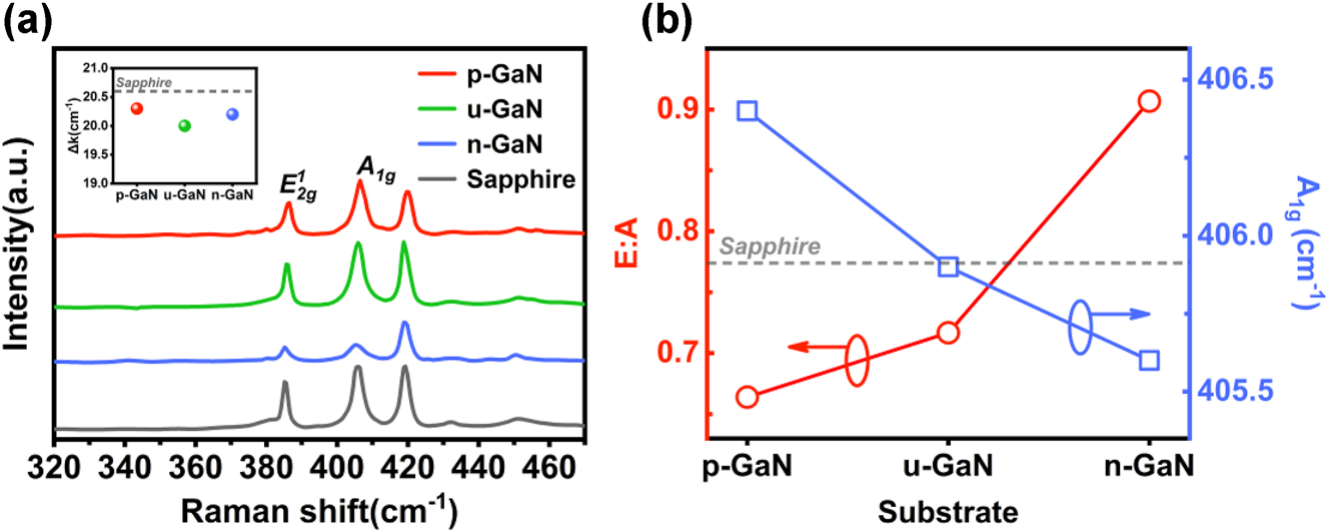
Effect of the differently doped GaN on the Raman modes of monolayer MoS_2_. (a) Raman spectra of monolayer MoS_2_ nanosheets on differently doped GaN and Sapphire. Inset is the frequency difference (Δ*k*) between $E_{2\text{g}}^{1}$ and *A*_1g_ modes of all samples. (b) Intensity ratio of the $E_{2\text{g}}^{1}$ and *A*_1g_ peak and the frequency of the *A*_1g_ mode as a function of the doping type of GaN.

According to previous reports, 2D layered MoS_2_ is sensitive to the surrounding environment, especially the substrates, that may dope the upper monolayer and thereby affect the exciton dynamics [43, 44]. To further reveal the doping effect and charge transfer properties of MoS_2_ in the heterostructures, monolayer MoS_2_ has been grown on differently doped GaN under the same condition and compared by Raman spectra. The optical resonance effects associated with the substrate’s geometrical features were largely ignored, since the structures were completely identical. Figure 3(a) depicts a comparison of the Raman spectra measured for monolayer MoS_2_ on differently doped GaN, in which the MoS_2_ on sapphire is also given as a reference. For the MoS_2_ on GaN, reduced frequency difference (Δ*k*) between two Raman characteristic modes is observed, accompanied with blue-shift of the ${\text{E}}_{2\text{g}}^{1}$ peak, which indicate less tensile strain in MoS_2_ due to perfect lattice matching [16, 45]. In addition, the Raman intensity ratio of the $E_{2\text{g}}^{1}$ to *A*_1g_ mode was further investigated in Figure 3(b), as it can be regarded as an indicator of doping levels [37, 46]. The observed lower ratio of monolayer MoS_2_ on unintentionally doped u-GaN than the counterparts on sapphire may be ascribed to the higher crystalline quality with fewer sulfur vacancies which generally behave as deep donors. Moreover, when the doping type of GaN is converted from n-type to *p*-type, the *E*: A ratio is largely reduced, suggesting a much lower n-doping level in MoS_2_. This modulation of the n-doping level of MoS_2_ can be mainly attributed to substrate-induced doping effect, which also results in the substantial blue-shift of *A*_1g_ mode [47].

**Figure 4:**
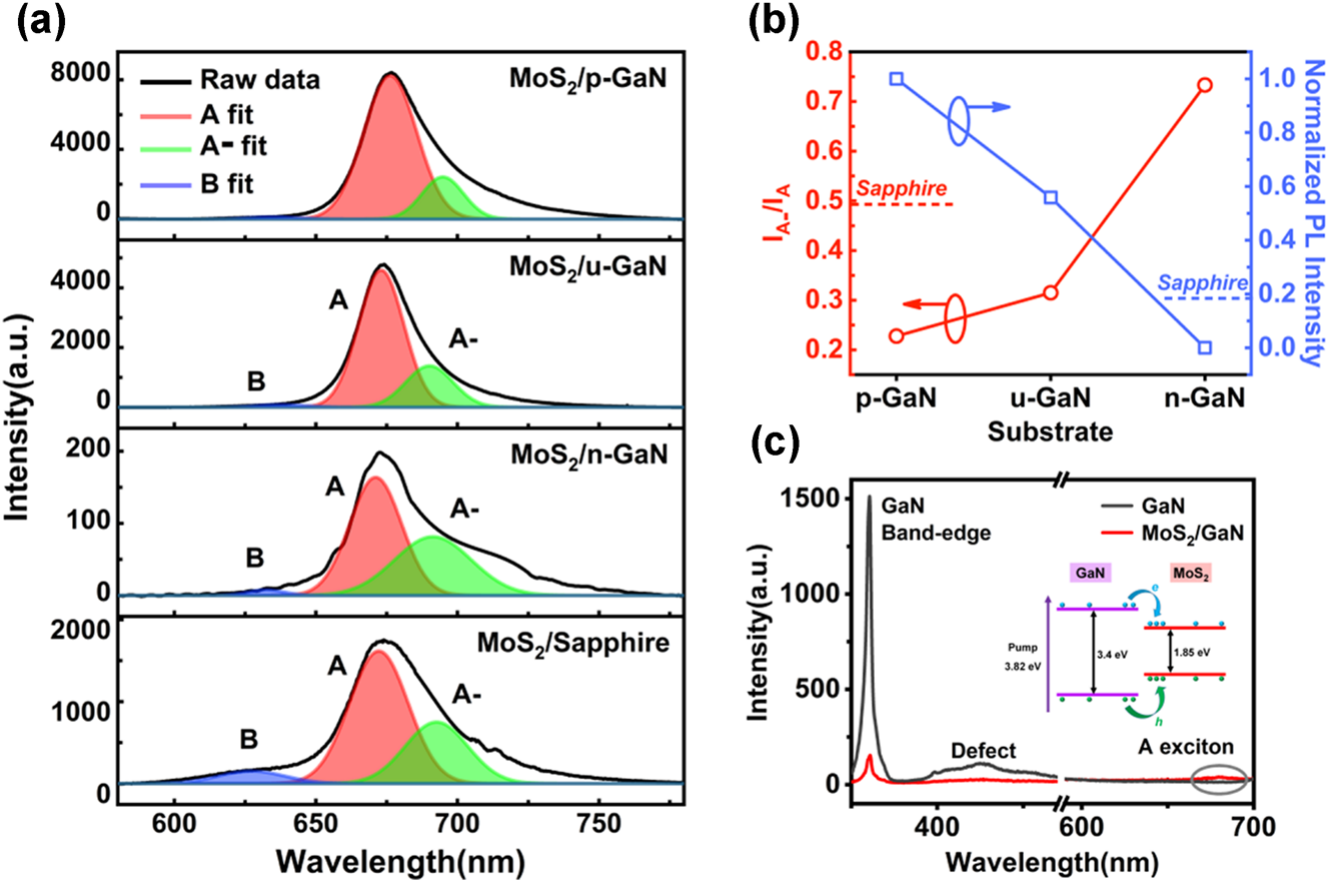
Effect of the differently doped GaN on the photoluminescence of monolayer MoS_2_. (a) PL spectra (excitation wavelength: 532 nm) and corresponding exciton peak fitting of monolayer MoS_2_ on p-GaN, u-GaN, n-GaN and sapphire. The neutral A exciton (red), B exciton (blue) and negative A-trion (green) fit are indicated together with the resulting cumulative spectrum (black). (b) The spectrum weight ratio *I*_A−_/*I*_A_ and normalized PL intensity as a function of the doping type of GaN. (c) PL spectra (excitation wavelength: 325 nm) of MoS_2_/u-GaN heterostructure and bare u-GaN. Inset shows the schematic of the charge transfer at the MoS_2_/GaN heterointerface under such excitation.

To confirm the above findings, the PL spectra (excitation wavelength: 532 nm) of the monolayer MoS_2_ on all substrates were further analyzed by Lorentzian fitting of the experimental spectra in order to extract the individual excitonic transitions. Figure 4(a) shows three fitted exciton peaks of the monolayer MoS_2_ on different substrates, which are identified as neutral A and B excitons as well as negative trions (A−). The two neutral exciton emission peaks are ascribed to the splitting of valence-band maximum owing to the broken spatial-inversion symmetry in monolayer MoS_2_, whereas the trions (A−) are formed by the binding of a free electron to a neutral A exciton, represented as e + A → A− [48]. It is noteworthy that a larger PL intensity ratio of A exciton versus B exciton (A/B) can be observed in MoS_2_ on GaN, illustrating the excellent quality, as this ratio is considered an effective method to evaluate the crystal quality of MoS_2_ qualitatively [49]. Besides, the PL of monolayer MoS_2_ on p-GaN substrate is strongest among all the substrates we studied, around five times higher than that on sapphire substrate, while that on n-GaN substrate is the worst. These features indicate that the MoS_2_ systhesized on these substrates are differently n-doped, which will modulate the relative concentration of trions and neutral excitons [44]. Specifically, this relative concentration can be further analyzed by spectrum weight ratio *I*_A−_/*I*_A_, as displayed in Figure 4(b), where the *I*_A_ and *I*_A−_ are the integrated emission intensity of neutral excitons and trions, respectively [50, 51]. As it can be seen that, the *I*_A−_/*I*_A_ ratios of MoS_2_ on p-GaN and u-GaN are much smaller than that on n-GaN, indicating the concentration of trions A-is lower in MoS_2_ on p-GaN and u-GaN. Therefore, it can be concluded that p-GaN can attract electrons from monolayer MoS_2_ and weaken its intrinsic n-doping while n-GaN provides electrons and enhance the n-doping level. Meanwhile, since trion emission has lower efficiency and broader spectrum compared to the exciton emission [44, 51], the normalized PL intensity of monolayer MoS_2_ decreases when the substrate changes from p-GaN, u-GaN to n-GaN.

To further elucidate the charge transfer process in MoS_2_/GaN heterostructures, different excitation sources were employed to excite GaN and MoS_2_/GaN heterostructures, respectively. Figure 4(c) displays the PL spectra of the MoS_2_/u-GaN heterostructures under the excitation of a 325 nm laser excitation. The PL spectrum of u-GaN consists of its band-edge emission centered at 360 nm as well as a weak defect emission centered around 450 nm. However, these two emissions are remarkably weakened in MoS_2_/u-GaN heterostructures, owing to the type I band alignment at the heterointerface, which facilitates the transfer of the photocarriers in GaN to monolayer MoS_2_ [16, 33]. More details about the band alignment of our samples are described in Supplementary Information (Note 1), based on the Kelvin probe force microscopy (KPFM) measurements (Figure S6), UPS spectra (Figure S7) and the established band diagram (Figure S8). Notably, the PL intensity of MoS_2_ is observed to be relatively weak, which can be attributed to the dominant non-radiative recombination of the hot carriers excited by such high photon energy [52]. In addition, the PL spectra of the heterostructures formed by MoS_2_ and other doped GaN is shown in Figure S9. Faint band-edge emission and defect emission of GaN in those heterostructures can also be observed, further verifying the efficient charge transfer process in MoS_2_/GaN heterostructures.

**Figure 5:**
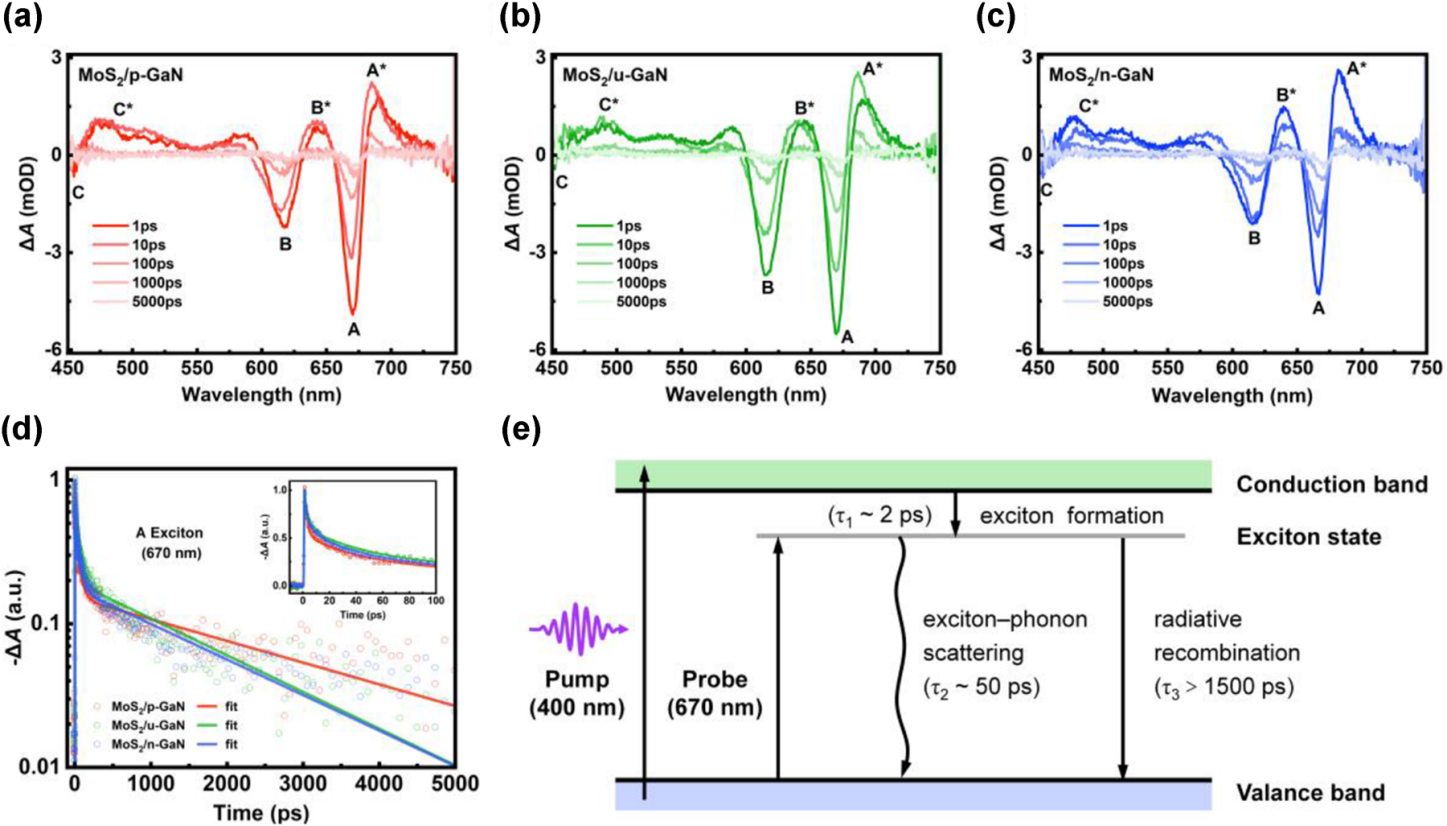
Effect of the differently doped GaN on the exciton dynamics of monolayer MoS_2_. (a–c) Transient absorption spectra of the monolayer MoS_2_ on p-GaN (a), u-GaN (b) and n-GaN (c) at different delay times, respectively, under excitations with fixed pump density of 100 μJ/cm^2^. (d) The kinetics traces of the A exciton state extracted from the TA results of samples with three doping types. The inset is a zoom-in view of the early 100 ps. The open circles are experimental data, the solid lines are the exponential fitting described in the main text, with consideration of the instrument response function (∼100 fs). (e) Schematic of the exciton dynamics in monolayer MoS_2_ on GaN.

To understand the ultrafast exciton dynamics in the MoS_2_ on differently doped GaN, pump-probe TA measurements were performed (details are provided in the Experimental Section). The transient absorption spectra decay from different samples after photoexcitation of 400 nm with density of 100 μJ/cm^2^ are depicted in Figure 5(a–c). The spectra of monolayer MoS_2_ on differently doped GaN at different pump–probe delay times all feature the same three prominent negative peaks A, B and C, which are ascribed to the ground-state bleaching (GSB) due to Pauli blocking or state filling of the excitonic resonances. The lower energy peaks A at ∼ 670 nm and B at ∼ 615 nm correspond to excitonic transitions located at the K point. The higher energy peak C near 450 nm can be assigned to band nesting transition in different regions of the Brillouin zone, especially at the Γ point [53, 54]. The two positive peaks A* (685 nm) and B* (640 nm), at the right side of bleaching peaks A and B, respectively, can be attributed to the band gap renormalization and broadening photoinduced absorption [53]. It is also worth mentioning that both A and B excitonic bands of MoS_2_ on GaN show a red-shift compared to those on sapphire (Figure S10), which could be caused by the exciton–phonon coupling in the heterostructures [16].

In Figure 5(d), we summarize the normalized kinetics of A exciton of the three samples extracted from the TA spectra, so that we are able to quantitatively analyze the dynamics. All relaxation dynamics exhibit a multi-exponential decay trend, and can be fitted well by a tri-exponential function of [55, 56]:

(1) $$S_{\text{A}}=A_{1}\exp\left(-\frac{t}{\tau_{1}}\right)+A_{2}\exp\left(-\frac{t}{\tau_{2}}\right)+A_{3}\exp\left(-\frac{t}{\tau_{3}}\right)$$

where *A*_1_, *A*_2_ and *A*_3_ represent the respective amplitude of lifetime *τ*_1_, *τ*_2,_ and *τ*_3_. The fitting parameters are tabulated in Table 1. The carrier relaxation processes for the A excitons are illustrated in Figure 5(e). The fast decay components (see the inset of Figure 5(e)) *τ*_1_ of ∼2 ps are assigned to the formation process of exciton from free electron-hole pairs under nonresonant excitation [57, 58], with consistent proportion of the whole decay process in a range of 40–50 % for all three samples. The slow components *τ*_2_ are in the same time scale of ∼50 ps for all samples with differently doped GaN substrates. The decay with *τ*_2_ may be attributed to the exciton–phonon scattering process, since the excitons generated in MoS_2_ are strongly coupled to GaN phonons via the 2D material–substrate interaction, then lose their energy to the crystal lattice by emitting phonons in GaN [16, 59]. Remarkably, the long decay components *τ*_3_ associated to the radiative recombination of excitons [56, 60], exist a strong correlation to the doping type of GaN. For the MoS_2_/p-GaN sample, the *τ*_3_ process takes 2800 ps, apparently longer than those in samples of MoS_2_/u-GaN (1756 ps) and MoS_2_/n-GaN (1680 ps). The prolonged exciton lifetime during radiative recombination in MoS_2_/p-GaN can be attributed to the increased concentration of exciton with longer lifetime and reduced concentration of trion with shorter lifetime caused by substrate doping, which is consistent with the lowest spectrum weight ratio *I*_A−_/*I*_A_ shown above [44].

**Table 1:** Fitting parameters of the kinetic traces of A-exciton decay (extracted at 670 nm) in MoS_2_/p-GaN, MoS_2_/u-GaN and MoS_2_/n-GaN heterostructures, under excitation of 400 nm.

Sample	*A*_1_	*τ*_1_ (ps)	*A*_2_	*τ*_2_ (ps)	*A*_3_	*τ*_3_ (ps)
MoS_2_/p-GaN	51.9 %	1.932	34.3 %	50.30	13.8 %	2873
MoS_2_/u-GaN	40.7 %	2.400	40.6 %	53.40	18.7 %	1730
MoS_2_/n-GaN	43.5 %	2.800	40.0 %	48.60	16.5 %	1656

## 4 Conclusions

In summary, we have successfully demonstrated a simple and cost-efficient method for large-scale fabrication of MoS_2_/GaN heterostructures by pre-spinning a mixed solution of Na_2_MoO_4_ and NaOH on GaN substrates followed by CVD sulfurization. Large triangle-shaped monolayer MoS_2_ single-crystal nanosheets with side length more than 400 μm were achieved on differently doped GaN, covering almost 90 % of the surface. Moreover, it is found that the doping type of GaN can significantly modulate the n-doping level of MoS_2_ via the substrate doping effect. The PL intensity is enhanced and the exciton radiative recombination lifetime is prolonged in monolayer MoS_2_ on p-GaN due to the increased exciton concentration and reduced trion concentration. Our work hereby opens a pathway for large-scale fabrication of MoS_2_/GaN heterostructures, as well as an insight into their charge transfer properties and exciton dynamics, which should be beneficial to develop optoelectronic devices based on this heterostructure.

## Supporting Information

Epitaxial structures of differently doped GaN; Schematic diagrams of MoS_2_ monolayer grown by our CVD method; additional results on XPS spectra of MoS_2_/GaN heterostructures; Optical microscopy images, KPFM measurements, UPS spectra, PL spectra and band diagrams of MoS_2_/p-GaN and MoS_2_/n-GaN heterostructures; Transient absorption spectra of the monolayer MoS_2_ on Sapphire.

## Supplementary Material

Supplementary Material Details
