# Supplementary material for: Large-scale synthesis and exciton dynamics of monolayer MoS2 on differently doped GaN substrates
Source: Nanophotonics. 2023 Nov 22;12(24):4475–84. doi: 10.1515/nanoph-2023-0503 (PMC11501315; doi:10.1515/nanoph-2023-0503)
Supplement: Supplementary file 1 — Supplementary Material Details [file j_nanoph-2023-0503_suppl_001.docx]

**Supporting Information**

**Large-scale synthesis and exciton dynamics of monolayer MoS_2_ on differently doped GaN substrates**

Pengcheng Jian, Xueqing Cai, Yongming Zhao, Dongyan Li, Zheng Zhang, Weijie Liu, Dan Xu, Wenxi Liang, Xing Zhou, Jiangnan Dai, Feng Wu* and Changqing Chen*

P. Jian, X. Cai, Y. Zhao, D. Li, Z. Zhang, W. Liu, D. Xu, Prof. W. Liang, Prof. X. Zhou, Prof. J. Dai, Prof. F. Wu, Prof. C. Chen

Wuhan National Laboratory for Optoelectronics

Huazhong University of Science and Technology

Wuhan 430074, China
E-mail: wufeng123@hust.edu.cn; [cqchen@hust.edu.cn](mailto:cqchen@hust.edu.cn)

D. Li, Prof. X. Zhou

State Key Laboratory of Materials Processing and Die & Mould Technology

School of Materials Science and Engineering

Huazhong University of Science and Technology

Wuhan 430074, China


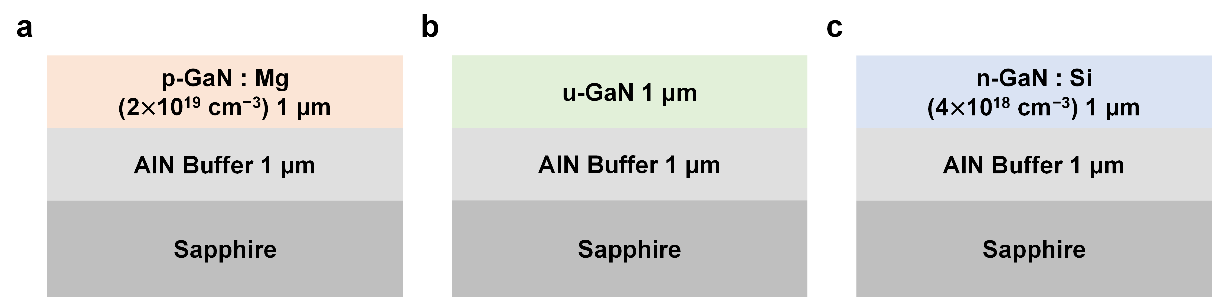


**Figure S1.** (a-c) The corresponding epitaxial structures of p-GaN (a), u-GaN (b), n-GaN (c) on Sapphire, respectively.


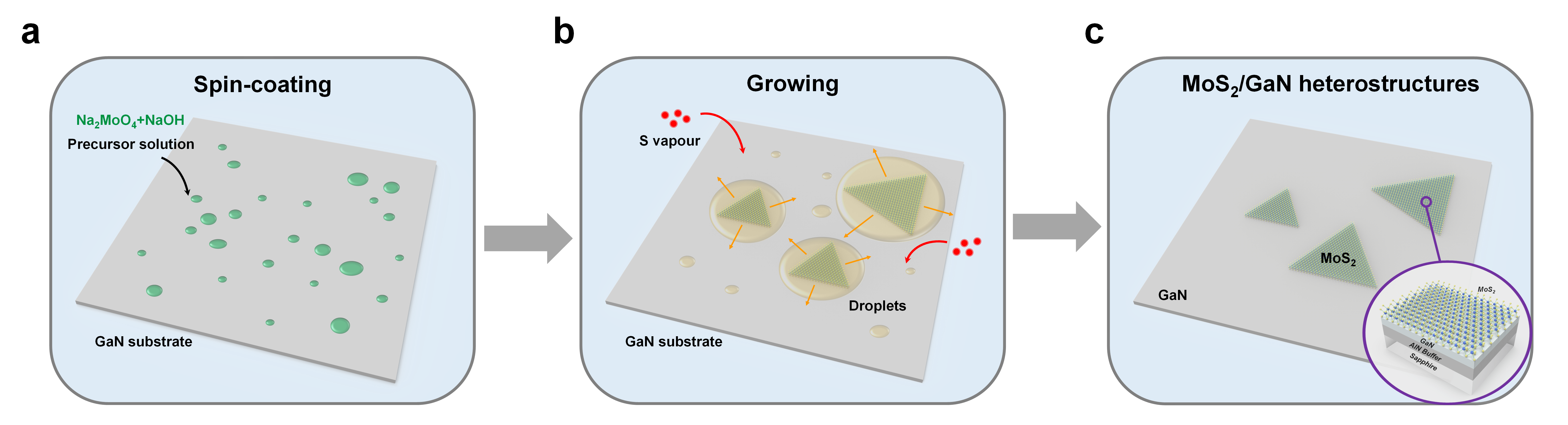


**Figure S2.** Schematic diagrams of MoS_2_ monolayer grown by NaOH assisted vapor-liquid-solid CVD method. (a) Liquid precursor (Na_2_MoO_4_+NaOH) on GaN substrate achieved by spin-coating process. (b) Liquid droplets form and adsorb sulfur vapor continuously. These Na-Mo-O-S droplets diffuse laterally on the substrate and precipitate out MoS_2_ when saturated. (c) Large size monolayer MoS_2_ obtained on the GaN. Inset: Enlarged view of the MoS_2_/GaN heterostructure.


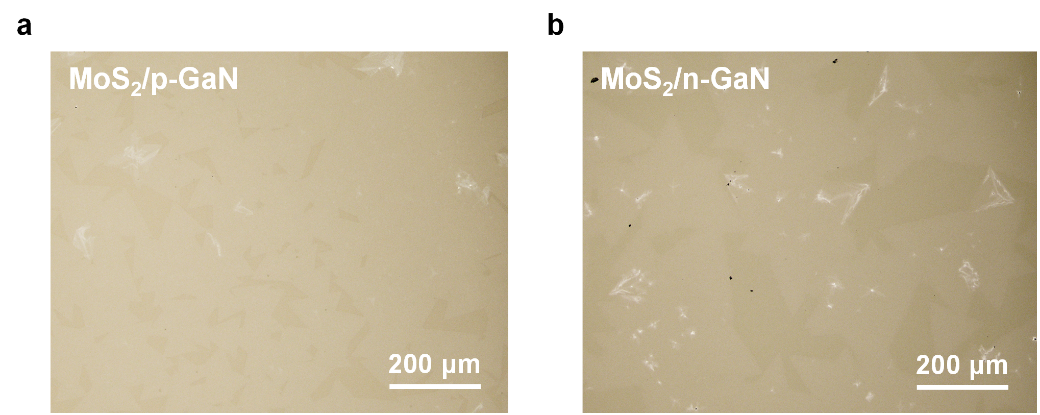


**Figure S3.** (a, b) Optical microscopy image of the as-grown MoS_2_ nanosheets on p-GaN (a) and n-GaN (b), respectively.





**Figure S4.** Photoluminescence spectra of monolayer MoS_2_ grown on GaN (red) and the substrate (grey), respectively. Two peaks located at 694 nm and 696 nm, respectively, are attributed to the radiation transition in impurity ions in sapphire.


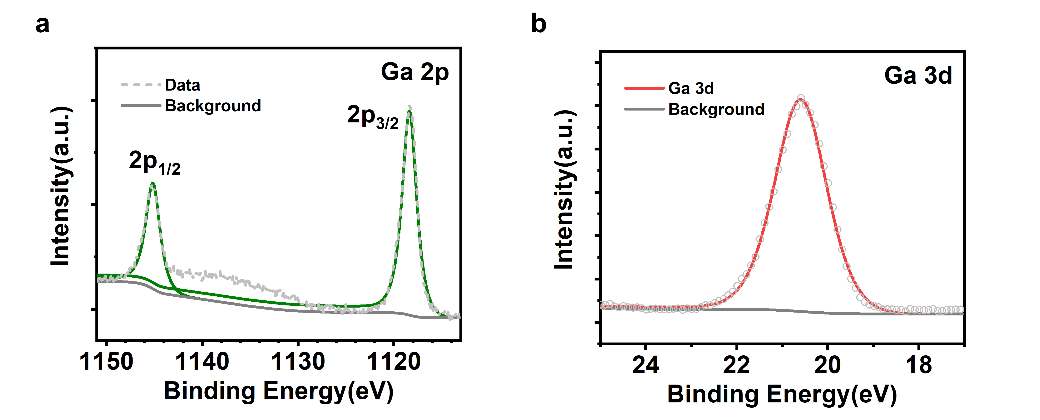


**Figure S5.** (a, b) Core level XPS spectra and peak fits from Ga 2p (a) and Ga 3d (b) regions for the MoS_2_/GaN heterostructure.

**Note 1:**

To gain insight into the band alignment of the MoS_2_/GaN heterostructure, the Kelvin Probe Force Microscopy (KPFM) measurements are necessary to be employed. Unfortunately, the sample on u-GaN is difficult to measure precisely due to the severe charge accumulation caused by poor conductivity of u-GaN. Thus, we hereby provide only the results of the samples on p-GaN and n-GaN, since their conductivity are much better. As shown in **Figure S6**a and c, the KPFM images display a clear surface potential contrast between monolayer MoS_2_ and differently doped GaN substrate. From the potential profile across the MoS_2_ edge (Figure R7b), we obtain a surface potential difference of ≈910 mV between monolayer MoS_2_ and p-GaN. For the sample of MoS_2_/n-GaN, however, the surface potential difference is of ≈-44 mV. After calibrating the tip work function using a gold thin film (+63 mV measured here) with a well-known work function of 5.10 eV, we are able to determine the work functions of p-GaN and n-GaN to be 6.17 eV and 4.96 eV, respectively. It is worth noting that the work functions of monolayer MoS_2_ on p-GaN and n-GaN are 5.16 eV and 5.00 eV, respectively, which agree well with our conclusion about the doping effect induced by the substrate (differently doped GaN).

Additionally, the positions of the Fermi level (E_f_) from the valence band maximum (VBM) for MoS_2_ in each heterostructure were further expressed from the UPS analysis, as depicted in **Figure S7**. In the same reason, the MoS_2_/u-GaN sample could not be analyze accurately by UPS spectra due to the poor conductivity. From Figure S7a and c, it was confirmed that the MoS_2_/p-GaN and the MoS_2_/n-GaN have E_f_ –VBM values of 1.54 eV and 1.66 eV, respectively. Besides, the calculated work function are 4.67 eV and 4.78 eV, respectively, for monolayer MoS_2_ on p-GaN and n-GaN, which is consistent with our KPFM results.

Based on the measured work function (φMoS_2_ (on p-GaN) = 5.16 eV; φp-GaN = 6.17 eV; φMoS_2_ (on n-GaN) = 5.00 eV; φn-GaN = 4.96 eV), calculated optical band gap (1.85 eV for MoS2 and 3.4 eV for GaN), UPS measurements, and reported electron affinity (χGaN = 4.1 eV), the band diagram of MoS_2_/p-GaN and MoS_2_/n-GaN can be established as **Figure S8**. Both heterostructures show straddling alignment (type I), which agrees well with the conclusions in our work and is consistent with the previous researches of monolayer MoS_2_ and GaN heterostructures.


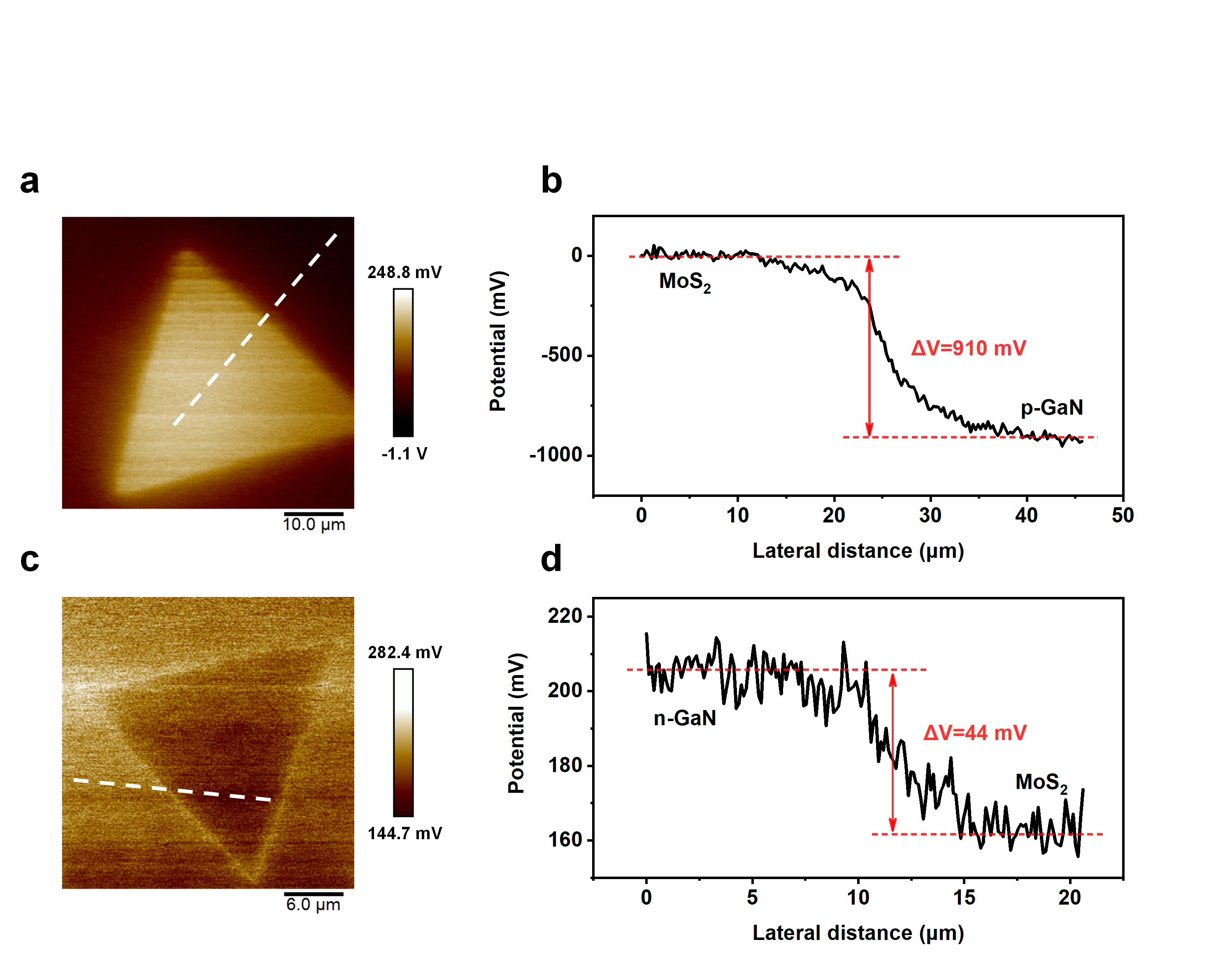


**Figure S6.** (a, c) KPFM images of monolayer MoS_2_ on p-GaN (a) and monolayer MoS_2_ on n-GaN (c). (b, d) Surface potential profile across the MoS_2_/p-GaN interface (b) and MoS_2_/n-GaN interface (d).


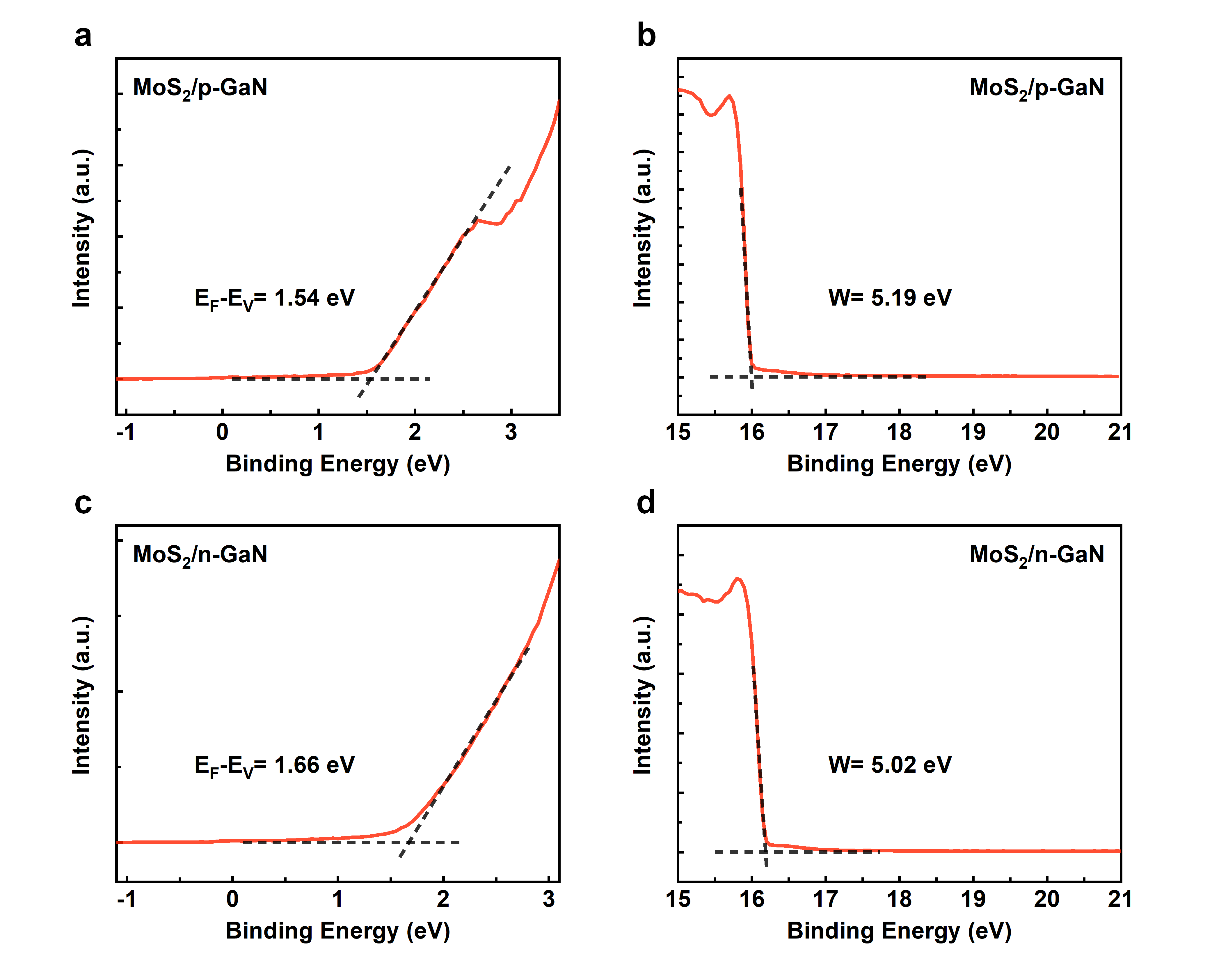


**Figure S7.** UPS spectra of MoS_2_/p-GaN and MoS_2_/n-GaN samples. (a, c) UPS spectra for the determination of E_f_-VBM position of MoS_2_ on p-GaN (a) and MoS_2_ on n-GaN (c). (b, d) UPS spectra for the determination of work function of MoS_2_ on p-GaN (b) and MoS_2_ on n-GaN (d).


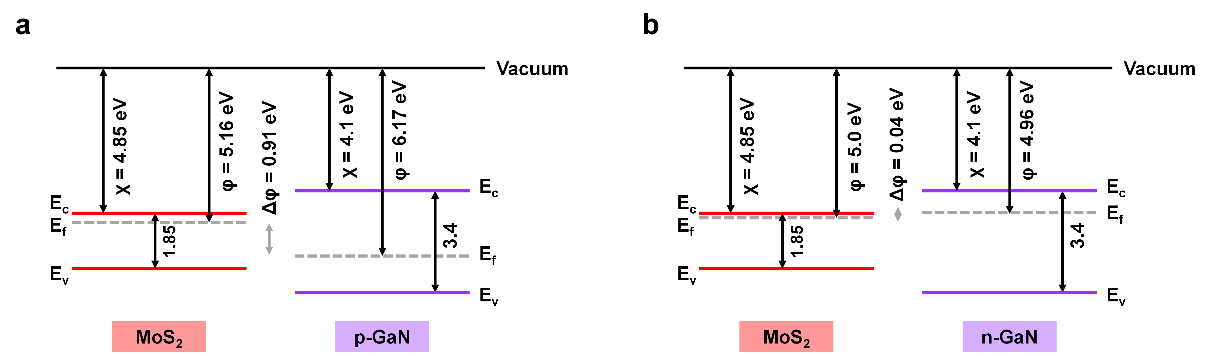


**Figure S8.** The band diagrams for MoS_2_/p-GaN and MoS_2_/n-GaN.


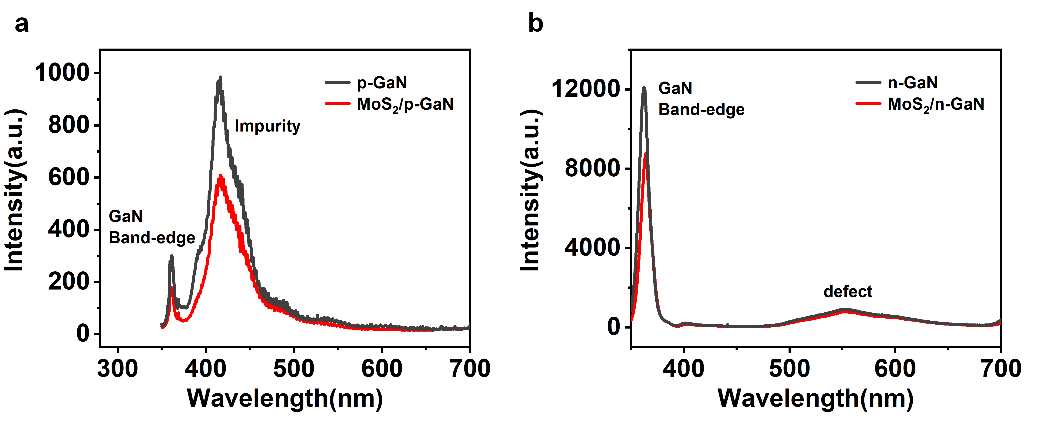


**Figure S9.** (a) PL spectra (excitation wavelength: 325 nm) of MoS_2_/p-GaN heterostructure and bare p-GaN. (b) PL spectra (excitation wavelength: 325 nm) of MoS_2_/n-GaN heterostructure and bare n-GaN.


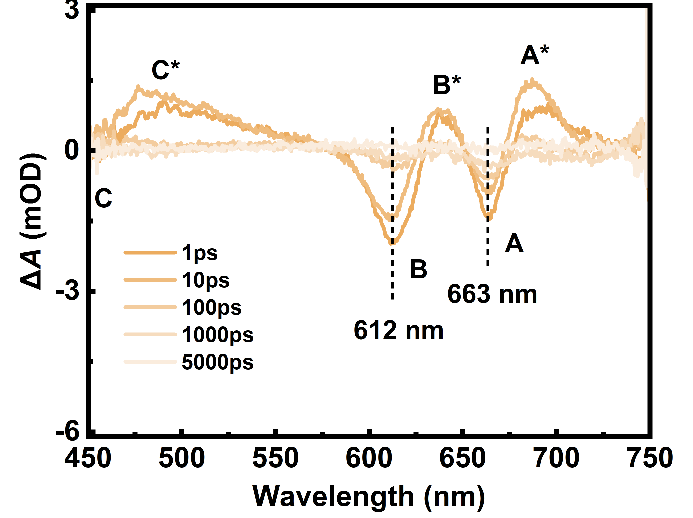


**Figure S10.** Transient absorption spectra of the monolayer MoS_2_ on Sapphire at different delay times under a fixed pump density of 100 μJ/cm^2^
